# Nurse-Led Mental Health Interventions for College Students: A Systematic Review

**DOI:** 10.5888/pcd22.240200

**Published:** 2025-05-01

**Authors:** Nancy G. Russell, Tamar Rodney, Jennifer K. Peterson, Andreea Baker, Lucine Francis

**Affiliations:** 1 Johns Hopkins University Student Health & Well-Being, Baltimore, Maryland; 2 Johns Hopkins University School of Nursing, Baltimore, Maryland; 3 Doane University College Health & Wellness, Crete, Nebraska; 4 Johns Hopkins University Center for School Health, Baltimore, Maryland

## Abstract

**Introduction:**

Mental health conditions such as anxiety, depression, and suicidal ideation or suicide are prevalent among college students and are a growing public health concern. The COVID-19 pandemic exacerbated this crisis. Nurses are a vital component of college health services and may be the first or only health care provider to evaluate college students experiencing a mental health condition. However, the literature has limited evidence on the nurse’s role and its impact on college mental health. Our systematic review examines nurse-led mental health interventions for college students.

**Methods:**

We conducted a comprehensive search for nurse-led interventions in college mental health by using PubMed, Embase, CINAHL, PsycInfo, and ERIC databases. A total of 2,814 articles were identified, and 2,290 were screened after removal of 524 duplicates. Thirty-five studies were reviewed for eligibility, and 16 were included in this review. The Johns Hopkins Nursing Evidence-Based Practice (JHNEBP) Hierarchy of Evidence Guide and Appraisal Tools were used to rate the strength and quality of the evidence, and the selected articles were generally of good quality.

**Results:**

The 16 articles came from institutions in North America (n = 5), southeastern Europe (n = 3), and Asia (n = 8). The studies focused on samples with a pre-existing mental health diagnosis or on mental health symptoms and interventions aimed at addressing mental health concerns. The interventions were facilitated by nurses and included various approaches, including experimental, quality improvement, and educational strategies.

**Conclusion:**

This review underscores the crucial role of nurses in addressing mental health issues among college students. Despite variability in approaches, nurse-led interventions offer promise in enhancing student well-being. Further studies are essential to gauge effectiveness and shape policies for supporting the nurse’s unique role in higher education. Integrating these findings into practice and policy will equip college health services to meet students’ evolving needs. Leveraging the expertise of nurses can enhance student mental well-being, leading to improved academic outcomes and overall quality of life.

**Summary**

**What is already known on this topic?**

College students are among those disproportionately affected by depression, anxiety, and suicidal thoughts, and nurses may be the first or only health care provider to evaluate a college student struggling with these mental health conditions. The role of nurses in mental health for college students is underreported in the literature.

**What is added by this report?**

This systematic review of 16 articles identified differences in college health nursing roles related to student mental health symptoms or diagnoses. Nursing interventions generally have a positive effect on college students’ mental health.

**What are the implications for public health practice?**

Nurses are an important source of evidence-based mental health care for college students. Future research is needed to further delineate nurse-led mental health support on college campuses.

## Introduction

Mental health conditions, including anxiety, depression, suicidal ideation, and suicide are global public health concerns and are highly prevalent among the college-aged population (1–4). Early adulthood and late adolescence are the periods in which 75% of mental health conditions begin, coinciding with the traditional college age for most students (5). Mental health symptoms are individual experiences or signs, such as persistent sadness or anxiety, that indicate a potential issue. A formal diagnosis, on the other hand, is a professional assessment made by a clinician based on a comprehensive evaluation of these symptoms and other criteria, leading to classification of a specific mental health condition, such as generalized anxiety disorder (6,7). Additionally, symptoms of different mental health diagnoses overlap. For example, symptoms such as sleep disturbances, irritability, and difficulty concentrating can appear in both depressive and anxiety disorders (6,7).

Suicide is the fourth leading cause of death worldwide among people aged 15 to 29 years (8). Over the last 3 decades, rates have been increasing among those aged 10 to 24 years in Central Europe, the US, the United Kingdom, Latin America, and Australasia (9). In the US, suicide is the second leading cause of death for people in the 10- to 24-year-old age group (3). US males have the highest age-standardized suicide rates globally (8,9). People who are adversely affected by social determinants of health, those from sexual minority communities, and those from tribal or indigenous groups have the highest rates of suicide (3,8). According to the American College Health Association (ACHA) National College Health Assessment (NCHA), which included more than 54,000 undergraduate student respondents, approximately 35% reported having anxiety, 27% reported having depression, 23% reported having both anxiety and depression, 8% reported having posttraumatic stress disorder (PTSD), 12% reported intentional self-harm, and 30% reported positive suicidal behavior, with 3% reporting a suicide attempt within the previous 12 months (4). The 2022 ACHA NCHA found that some students experience higher rates of anxiety, depression, PTSD, intentional self-harm, suicidal behavior, and suicide attempts compared to others (4). Mental health conditions affect academic performance (10,11) and potentially overall progression and outcomes (10,12) and are associated with other adverse health and social effects (12). College student mental health concerns are increasing in prevalence, and the COVID-19 pandemic has further exacerbated this mental health crisis, particularly with disruptions to lifestyle, loss of social support, risk of illness, and isolation (13). The prevalence of mental health conditions among college students and the lifelong impact of these conditions make mental health a crucially important aspect of college health.

Emphasis on college health and associated programming has expanded substantially since its development in the US in the late nineteenth century (14). Today, college health programs vary widely, with some institutions providing only nursing services and others providing a full array of integrated well- and sick-care services (15). College health is a focus on advancing the health, wellness, and well-being of college students and campus communities (16). However, “college health” is not consistently defined, which highlights its understudied aspects and the roles of college health providers globally. Furthermore, the demand for mental health services on college campuses, already high before the COVID-19 pandemic, is even higher postpandemic, due in part to increased levels of need for student mental health support (17).

Nurses are a substantial portion of a college health center’s clinician staff. According to ACHA (18), of the 62 US institutions that completed the Institutional Profile Survey data survey for the academic year 2022–2023, these institutions employed approximately 80 psychiatric nurse practitioners, 443 primary care nurse practitioners, 862 registered nurses, 158 licensed practical nurses and licensed vocational nurses, and 33 certified nursing assistants. College health centers may also be nurse-directed, where the primary administrative director is a registered nurse or an advanced practice nurse (19). ACHA found that among US college health centers that merged or integrated counseling and health services, nearly 30% reported the professional affiliation of the center director as a nurse (20).

Nurses play an important role in student well-being and academic success (19,21). Nurses may serve as the first or only health care professional that students seek out for support and care, including for a mental health crisis or mental health evaluation, on a college campus (19,20). Nurses can also play a key role in developing and implementing interventions to improve the mental health of college students (22), including mental health screenings and surveillance. By offering mental health screenings and interventions through college health services, students can obtain timely assessment, evaluation, and treatment of their mental health conditions, including referrals to other campus or community partners (eg, counselors, psychiatrists) or other campus resources (eg, academic support services).

The role of nurses in providing mental health assessment and care in college settings is understudied, despite the mental health crisis affecting this population. This systematic review examines nurse-led mental health interventions for college students.

## Methods

### Data sources

We used the PRISMA (Preferred Reporting Items for Systematic Reviews and Meta-Analyses) standards for reporting the results of this systematic review (23). In collaboration with an experienced clinical informationist and librarian, we built a search strategy and retrieved citations from the following databases: PubMed, Embase, CINAHL, APA PsycInfo, and ERIC (Appendix). We did not include reviews. The search was completed on January 5, 2024. As an example, we included MeSH (Medical Subject Headings) terms and key words that included “student health services” OR “universities,” OR “college student” and “anxiety” OR “depression” and “nurses”” OR “nurse led.” The search results were imported into Covidence (Veritas Health Innovation), an online software, to facilitate the conduct of the systematic review. We included studies from settings across the globe that were published in English from 2015 to 2024 in peer-reviewed journals. The college student population was not defined by age because of variation in college student demographics. Exclusion criteria included no full text available in English, gray literature, or non–peer-reviewed publications. We defined “nurse-led” as nurse participation in authorship or the design, implementation, or evaluation of the intervention to capture information on various degrees of nurse involvement. This criterion was used during full-text review. We defined “mental health conditions” as a diagnosis or symptoms of mood disorders that are prevalent in the college-aged population, such as anxiety, depression, or suicidal ideation.

### Study selection

Two independent reviewers (N.G.R., J.K.P.) screened titles and abstracts to determine eligibility for full-text review. Differences between the 2 reviewers were resolved through discussion. Articles eligible for full-text review were retrieved in PDF format and imported into Covidence, and 2 authors (N.G.R.,T.R.) conducted full-text review to identify articles for inclusion in the review. Disagreements were resolved through group discussion. A PRISMA flow diagram was constructed to describe study screening and selection (Figure).

**Figure:**
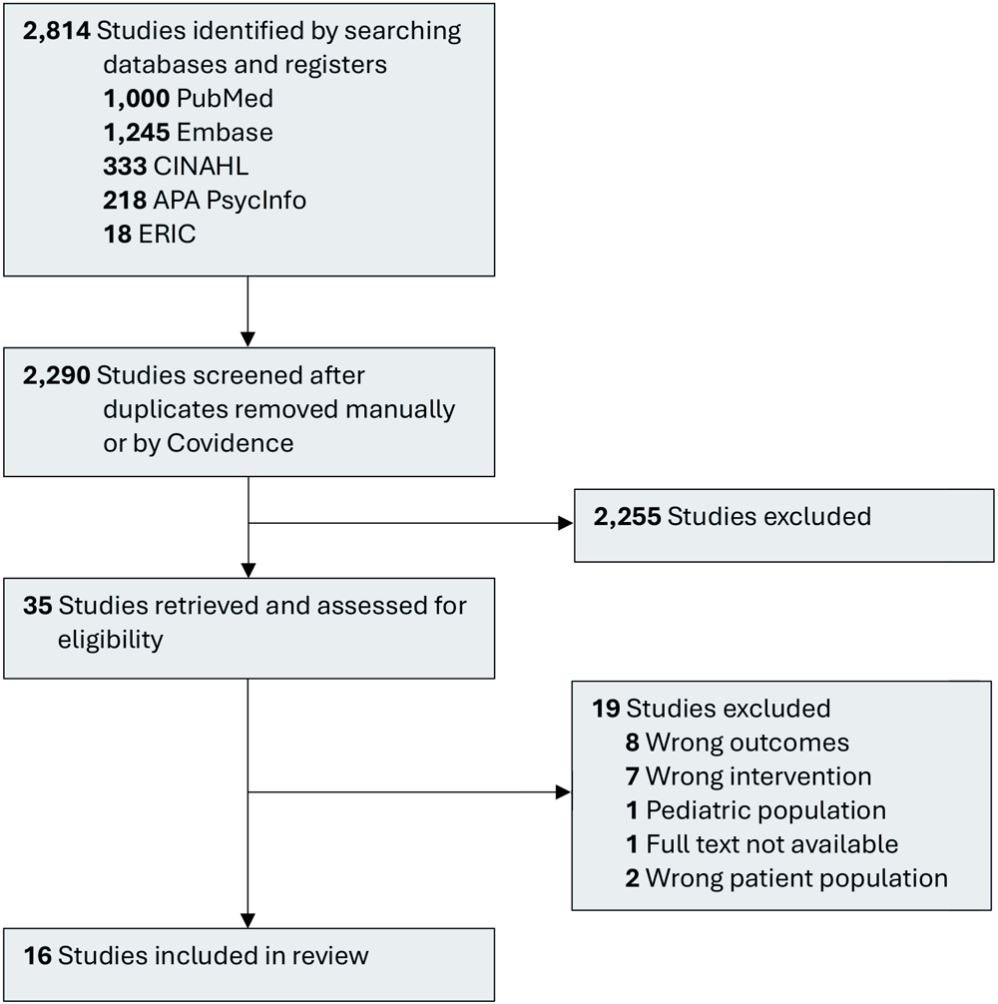
PRISMA flow chart: nurse-led mental health interventions for college students. *From:*The PRISMA 2020 statement: an updated guideline for reporting systematic reviews (23).

### Data extraction

Two reviewers (N.G.R., T.R.) abstracted the remaining articles independently. The group met weekly to discuss progress and resolve questions about articles. The abstracted data included citation information, setting, study demographic characteristics, intervention description, role of nursing, and results. The articles were also categorized as to whether participants in the described studies had an active mental health diagnosis or were symptomatic without a formal diagnosis. After the results were discussed, the data were synthesized. We developed an a priori organizing framework which included the study’s purpose statement, study design, intervention description, participant demographic characteristics, and outcomes.

### Quality appraisal

The Johns Hopkins Nursing Evidence-Based Practice (JHNEBP) Hierarchy of Evidence Guide and Appraisal Tools were used to rate the strength and quality of the evidence (24). Developed by nurses initially for nurses (24), the JHNEBP model is a commonly used EBP model (25) that uniquely incorporates the evaluation of research and nonresearch evidence by level (levels I through III are research evidence, levels IV and V are nonresearch evidence) and quality (grades A through C, where A is high, B is good, and C is low) for an overall assessment of strength (24). This model was a strong fit for this review, which explored nurse-led mental health interventions for college students.

## Results

The final synthesis incorporated 16 studies conducted at institutions across the globe, including in North America, southeastern Europe, and Asia (26–41). These studies used diverse interventions aimed at addressing mental health concerns, encompassing a blend of quality improvement, intervention, and educational strategies. Each study included met our definition of nurse-led, with nurses implementing the intervention in most studies. An intriguing observation was that some studies included participants with pre-existing confirmed diagnoses, while others concentrated on symptoms (Table).

**Table:** Evidence Found in Systematic Review of Nurse-Led Mental Health Interventions for College Students

Purpose/aims	Study design	Intervention, nursing role	Setting, sample	Evidence strength, quality	Outcomes, instruments	Results
Aoki et al, 2019 (30 ), Japan						
Evaluate effect of a 7-day shared decision-making program on patient-perceived involvement Compare: • Overall satisfaction • Consultation times • Persistence of treatment • Medication adherence • Depression severity	RCT	Intervention: Shared Decision-Making Program • 3 steps • Decision coaching with a nurse and clinician Nursing role: Nurse-delivered shared decision coaching	Setting: Outpatient service of the health support center of 1 university Sample: • 88 undergraduate and postgraduate students (35 participants in intervention arm, 53 participants in control arm) diagnosed with a major depressive episode on QIDS-SR at baseline • 42.9% female • Average age 21.8 years	Level I, quality A	Primary outcome: Change in patient perceived involvement in medical decisions measured with the Combined Outcome Measure for Risk Communication and Treatment Decision-making Effectiveness tool Secondary outcomes: • Satisfaction with consultation duration • Whether participants looked up treatments and shared information • Severity of depressive symptoms measured with the QIDS-SR	Intervention group had significantly higher patient-perceived involvement in medical decision making. No significant differences noted in: • Overall satisfaction with care • Duration of consultation • Discussion of treatments • Looking up treatments • Medication adherence • Depression symptoms

Bever and Maks, 2023 (26), US						
Evaluate effect of a depression screening and referral protocol on depression screening and management in college students	QI: retrospective medical record review	Intervention: Depression screening and referral protocol implemented with PHQ-2, reflex to PHQ-9 and evaluation Nursing role: Nurses delivered screening and evaluation	Setting: 1 state university student health clinic Sample: 304 students presenting for a non–mental health visit	Level V, quality A	Primary outcome: Percentage of visits in which the PHQ-2 and PHQ-9 were administered and students were evaluated and referred and appropriately assessed with descriptive statistics	Increases in number and percentage of students screened and in students referred: 98.0% of students in project received PHQ-2. Of those with positive PHQ-2 scores, 71.4% completed PHQ-9. Of those with scores of ≤10 on PHQ-9, 93.3% were referred; 19.1% screened positive for suicide ideation, and provider addressed this immediately 100% of the time

Çekiç and Ançel, 2022 (31), Turkey						
Evaluate IRRA’s effect of on nursing students’ depressive symptoms and coping styles	RCT	Intervention: IRRA sessions Nursing role: IRRA sessions chaired and led by nurses	Setting: 2 state universities Sample: • 20 second-year nursing students with mild to moderate depression scores on BDI at baseline • 70% female • Average age 20.5 years	Level I, quality B	Primary outcomes: • Change in depression assessed with BDI • Change in Coping Styles Inventory scores	Intervention group had significantly decreased BDI scores; no significant difference in coping styles

Chen et al, 2015 (32), Taiwan						
Evaluate effect of Chinese 5-element music therapy on nursing students with depressed mood	2 group, experimental	Intervention: Chinese 5-element music therapy Nursing role: Nurse- administered music therapy	Setting: 1 nursing college Sample • 71 nursing students with depressed mood on DMSRIA at baseline • ~97% female • Average age, 18.5 years	Level I, quality A	Primary outcomes: • Change in depressed mood assessed with DMSRIA • Change in salivary cortisol levels	Intervention group with significantly decreased depression; nonsignificant reduction in salivary cortisol levels

Chen et al, 2022 (27), US						
Evaluate effect of a nurse-led self-management intervention on pain, symptom management, and quality of life in young adults with IBS	RCT	Intervention: Nurse-led one-to-one consultation plus a self-management online education program versus online education module program Nursing role: Nurse delivered consultation intervention	Setting: 2 public university campuses and 2 gastrointestinalclinics in 2 hospitals; intervention delivered in a research laboratory affiliated with a university-sponsored pain research center on the 2 university campuses Sample: • 80 young adults, with 39 in the nurse-led intervention group (69.2% of the intervention group were current college students) • 82.0% female • Age range 18–29 years • Average age 20–21 years	Level I, quality A	Primary outcomes: • Average pain intensity and pain interference assessed with the Brief Pain Inventory • IBS quality of life assessed with an IBS quality-of-life instrument • IBS-related symptoms (anxiety, depression, fatigue, sleep disturbance) assessed with the NIH PROMIS • Self-efficacy assessed with the Self-Efficacy for Managing Chronic Disease scale • Coping strategies assessed with the Coping Strategies Questionnaire-Revised (CSQ-R)	• Nurse-led intervention group with significantly improved anxiety • Both intervention groups with significantly improved IBS quality of life (dysphoria, health worry, relationship), average pain intensity and pain interference • Nurse-led intervention group with significantly greater improvement in quality of life than the online module intervention

Cho and Jang, 2021 (33), South Korea						
Evaluate effect of logo-autobiography for college students (LAC) on their stress and depression during the COVID-19 pandemic	Quasi-experimental	Intervention: LAC group sessions Nursing role: Nurse developed and delivered the LAC	Setting: 4 colleges in 2 cities; intervention delivered in a university seminar room Sample • 48 four-year students • 72.9% female • Average age 21.5 years	Level II, quality A	Primary outcomes: • Change in stress level assessed with the Perceived Stress Scale • Change in depressive symptoms assessed with the Center for Epidemiologic Studies Depression Scale-10 • Change in meaning of life assessed with the Purpose-In-Life (PIL-K) scale	Intervention group with significantly decreased depression and stress levels, and significantly increased meaning of life scores postintervention, with effects on stress and meaning of life continuing at 4 weeks postintervention.

Chueh et al, 2018 (34), Taiwan						
Evaluate effect of an auricular acupressure program on sleep quality, anxiousness, and depressed moods in nursing students with sleep disturbance	Quasi-experimental	Intervention: Auricular acupressure that applied a magnetic pellet on the shenmen acupoint Nursing role: Nurse performed the auricular acupressure intervention	Setting: 1 university intervention delivered in a nursing classroom setting Sample: • 36 nursing RN-BSN students who were working full-time in the hospital at the time of the study, with active sleep disturbance on Pittsburg Sleep Quality Index at baseline • 100% female • Average age 32 years	Level II, quality B	Primary outcomes: • Change in sleep quality assessed with the Pittsburg Sleep Quality Index • Change in anxiety assessed with the BDI • Change in depression assessed with the BDI-II	Intervention group had significant improvements in sleep quality, anxiety, and depressed mood

Falsafi, 2016 (28), US						
Evaluate effect of mindfulness and yoga in mitigating the effects of depression and/or anxiety in college students	RCT	Intervention: Training in mindfulness or yoga Nursing role: Nurse delivered intervention	Setting: 1 public university Sample: • 67 undergraduate students diagnosed with anxiety and/or depression • 86.4% female • Average age 22.1 years	Level I, quality A	Primary outcomes: • Change in depression assessed with the BDI • Change in anxiety assessed with the Hamilton Anxiety Scale • Change in stress assessed with the Student-Life Stress Inventory • Change in self-compassion assessed with the Self-Compassion Scale • CAMS–R	Intervention groups (mindfulness and yoga) with significant improvement in depression, anxiety, stress and mindfulness Significant change in self-compassion scores in mindfulness group only

Guo et al, 2017 (35), China						
Evaluate effect of a group positive psychotherapy (PPT) program on depression and self-efficacy in nursing students	RCT	Intervention: Group PPT program Nursing role: Nurse delivered the PPT intervention	Setting: 3 universities in the same province Sample: • 76 nursing undergraduate students with mild depression on BDI-II at baseline • 94.9% female • Average age 20.4 years	Level I, quality A	Primary outcomes: • Change in depression assessed with the BDI I-II • Change in self-efficacy assessed with the General Self-Efficacy Scale	Intervention group had significantly alleviated depression and significantly improved self-efficacy

Kaplan and Ançel, 2021 (36), Turkey						
Evaluate effect of IRRA on nursing student anxiety level and interpersonal problem-solving orientations	RCT	Intervention: IRRA sessions Nursing role: IRRA sessions led by nurses	Setting: 1 university’s nursing department Sample: • 16 sophomore nursing students with mild-moderate scores on BAI at baseline • 62.5% female • Average age 19.2 years	Level I, quality B	Primary outcomes: • Change in anxiety assessed with the BAI • Change in interpersonal problem-solving assessed with the Interpersonal Problem-Solving Inventory	Intervention group had significant improvements in anxiety and interpersonal problem-solving orientations

Khan et al, 2023 (37), Canada						
Evaluate effect of a health education intervention on self-rated health knowledge, levels of stress and anxiety, and ability to find and access school resources for international students	Pre/post	Intervention: Structured, 2-visit educational intervention Nursing role: Nurse delivered structured education at the campus health center	Setting: 1 college and 1 university on a shared campus Sample: • 202 international undergraduate and graduate students • 54% female • Average age 25 years	Level III, quality B	Primary outcomes: Change in: • Health knowledge • Levels of stress • Levels of anxiety • Ability to find resources • Ability to access resources All assessed with self-report survey	Postintervention had • Significant increases in student ability to get help for a mental health issue • Significant decreases in stress/anxiety • Significant increases in finding and accessing school resources

Kim and Lee, 2023 (38), South Korea						
Evaluate effect of an online mental health promotion program on posttraumatic stress, depression, functional health and college adaptation in traumatized female college students	RCT	Intervention: Online mental health promotion program Nursing role: Intervention program developed and led by nurses	Setting: 8 universities; intervention delivered via messaging application/email Sample • 34 students with a qualifying traumatic experience per the Stressful Life Events Screening Questionnaire (SLESQ) and nonsevere PTSD per the IES-R at baseline • 100% female • Age range 19–29 years	Level I, quality A	Primary outcomes: • Change in posttraumatic stress assessed with the Impact of Event Scale–Revised (IES–R) • Change in depression assessed with the Center for Epidemiological Studies Depression Scale • Change in functional health assessed with the Functional Health Pattern Assessment Screening Tool • Change in college adaptation assessed with the Student Adjustment to College Questionnaire	Intervention group had a significant decrease in posttraumatic stress and depression symptoms Nonsignificant improvements in functional health and adaptation to college life

Lee and Lee, 2020 (39), South Korea						
Explore effect of a group cognitive behavioral program on depression, self-esteem and interpersonal relations among undergraduate students	Quasi-experimental	Intervention: Group cognitive behavioral group program Nursing role: Program developed and led by nurses	Setting 1 university Sample • 37 undergraduate students • >70% female • Average age 22.1 years	Level II, quality A	Primary outcomes: • Change in depression assessed with the BDI • Change in self-esteem assessed with the Rosenberg the Self-Esteem Scale • Change in interpersonal relationship assessed with the Relationship Change Scale	Intervention group had significant improvements in depression, self-esteem, and interpersonal relationship

Şener Çetin and Şolt Kirca, 2023 (41), Turkey						
Evaluate effect of a mindfulness-based stress reduction program in decreasing premenstrual symptoms	RCT	Intervention: mindfulness-based stress reduction program provided in 8 weekly sessions lasting 2.5 hours and a 6-hour silence retreat during week 6 Administered via Zoom Nursing role: Nurse delivered intervention	Setting: 1 university’s midwifery department Sample: • 74 midwifery university students with a diagnosis of untreated PMS on PMSS at baseline • 100% female • Average age 21.0 years	Level I, quality A	Primary outcome: Change in PMS assessed with the PMSS	Intervention group had significantly improved PMS symptoms (including depression, anxiety, sleep changes, irritability, fatigue, pain, appetite changes) No significant change in PMS swelling

Slabaugh et al, 2018 (29), US						
Implement a quality improvement project for standardized screening and referral of depressive symptoms and identify factors related to mentoring program interest in a college health clinic	QI	Intervention: PHQ-2 and PHQ-9 were distributed with immediate evaluation as indicated Nursing role: Nurses delivered screening and evaluation	Setting: 1 university student health clinic Sample: • 160 university students not actively receiving treatment for depression • Age range 18–22 years	Level V, quality A	Primary outcome: Implementation of depression screening and referral assessed with PHQ-2, PHQ-9, and nurse intervention descriptive statistics	• Standardized depression screening and referral process successfully implemented • 165 PHQ-2 screens were completed • 8 completed PHQ-9 based on positive PHQ-2 scores • 7 received intervention for positive PHQ-9 scores

Tay, 2022 (40), Singapore						
Evaluate effect of the online HOPE intervention on help-seeking attitudes and intentions among university students	RCT	Intervention: HOPE – an online mental health educational intervention Nursing role: Nurse developed the intervention	Setting: 1 university; intervention delivered virtually Sample: • 174 university students • 71.6% female • Age range 18–24 years	Level I, quality A	Primary outcome: Self-report surveys to measure: • Recognition of depression • Barriers of help-seeking • Help-seeking intentions • Attitudes about interventions, help sources, medications • Perceptions about the intervention	• Intervention group had significantly lower acknowledgment of depression as stress • Significant improvement in help-seeking over time • Nonsignificant increases in acknowledgment of antidepressants, tranquilizers, and antipsychotics • No significant differences in barriers to help-seeking

Abbreviations: BAI, Beck Anxiety Inventory; BDI, Beck Depression Inventory; CAMS–R, Cognitive and Affective Mindfulness Scale–Revised; CSI, Coping Styles Inventory; DMSRIA, Depression Mood Self-Report Inventory for Adolescence; IBS, irritable bowel syndrome; IRRA; interpersonal relational role analysis; PHQ, Patient Health Questionnaire; PROMIS, Patient-Reported Outcomes Measurement Information System; NIH, National Institutes of Health; PMS, premenstrual syndrome; PMSS, Premenstrual Syndrome Scale; PTSD, posttraumatic stress disorder; QIDS-SR, Quick Inventory of Depressive Symptomatology–Self-Report; RCT, randomized controlled trial.

### Strength and quality rating

Of the 16 articles, 10 were rated as Level I evidence (27,28,30–32,35,36,38,40,41), 3 were rated as Level II evidence (33,34,39), 1 was rated as Level III evidence (37), and 2 were rated as Level V evidence (26,29). Most of the articles were rated as Level A High Quality Evidence (26–30,32,33,35,38–41), and 4 were rated as Level B Good Quality Evidence (31,34,36,37) (Table).

### Articles that focused on a mental health diagnosis

Six articles in our review included a sample of college students with a suspected or established mental health diagnosis, including major depressive disorder (30), depressive phase of bipolar disorder (30), depression (28,35,38,39), PTSD (38), and irritable bowel syndrome (IBS)–related mental health symptoms (27). Patients with IBS have a high prevalence and risk of psychiatric disturbance (42).

Four studies were conducted at institutions in Japan (30), China (35), and South Korea (38,39), and 2 studies were conducted at US institutions in Connecticut (27) and North Carolina (28). Four of these 6 studies included 1 college or university site (27,28,30,39), 1 included 3 sites (35), and 1 included 8 sites (38). Most articles included a broad sample of college and university students with a known mental health diagnosis (27,28,30,38,39), and 1 included only nursing students with a known mental health diagnosis (35).

**Intervention type**. The interventions used in the 6 studies in Japan, China, and South Korea exhibited a wide range of approaches. These included a shared decision-making program (30), an 8-week group positive psychotherapy (PPT) program (35), an eight-session online mental health program delivered through blog posts (38), a group cognitive behavioral program (39), an 8-week comparison of mindfulness and yoga intervention practices (28), and 10 video modules in a nurse-led online program, complemented by nurse-led one-to-one consultations as part of a nurse-led self-management program (27).

**Measurement tools**. Various measurement tools were used in the 6 studies, with the Beck Depression Inventory (BDI) being the most common (28,39), including the BDI-II (35). Other tools used to measure depression included the Quick Inventory of Depressive Symptomatology (QIDS-SR) (30) and the Center for Epidemiological Studies Depression Scale (35,38). Self-efficacy was measured with the General Self-Efficacy Scale (35) and the Self-Efficacy for Managing Chronic Disease tool (27). The Stressful Life Events Screening Questionnaire (38), Impact of Event Scale–Revised (38), Student Adjustment to College Questionnaire (38), and the Student Life Stress Inventory (28) were used to measure stress. Anxiety, mindfulness, self-compassion (28), self-esteem (39), relationship change (39), coping strategies (27), patient-perceived involvement in medical decisions (30), and functional health (38) were also measured. Chen and colleagues (27) used the brief pain inventory, the IBS quality of life scale, and the National Institutes of Health Patient-Reported Outcomes Measurement Information System for IBS-related symptoms, including anxiety, depression, fatigue, and sleep disturbance. Descriptive statistics and participant self-report were included as measurement tools (30).

**Outcomes**. Overall, significant improvements in mental health were noted in the 6 studies that included college student samples with a known mental health disorder. Four studies found a significant reduction in depression post-intervention (28,35,38,39), and 3 studies found a significant decrease in anxiety (27,28), stress (28), PTSD (38), and/or dysphoria (27). Significant improvements were also noted in self-efficacy (35), self-esteem (39), self-compassion and mindfulness (28), patient-perceived involvement in medical decision-making (30), and pain (27). Nonsignificant findings included improved functional health and adjustment to college life (38), and self-efficacy (27).

### Articles that focused on mental health symptoms

Ten articles in our review addressed symptoms experienced by college students, including depression (26,29,31–34,40), stress (33,37), sleep difficulty, which is associated with psychological distress (43), and anxiety (34,36), and premenstrual symptoms (41), which commonly include behavioral and mood disturbances that may exist alone or in conjunction with somatic symptoms (41,44,45).

Eight studies addressing symptoms in this review were conducted at colleges or universities in the following countries: Canada (37), Korea (33), Singapore (40), Turkey (31,36,41) and Taiwan (32,34). Two studies addressing symptoms were conducted at US institutions in Minnesota (26) and Pennsylvania (29). Seven studies were conducted at a single college or university site (26,29,32,34,36,40,41), 2 studies included 2 colleges or universities (31,37), and 1 study included 4 colleges or universities (33). Nursing students constituted the sample in 4 of these studies (31,32,34,36), and the other 6 studies included a broader sample of college and university student types (26,29,33,37,40,41).

**Intervention type**. Various interventions were used in these 10 studies, with some commonalities noted (26,29,31–34,36,37,40,41). Two studies implemented depression screening and referral protocols in college health centers (26,29). Educational interventions were used in 2 studies (37,40), and 2 other studies employed interpersonal relational role analysis (IRRA) interventions (31,36). Other interventions included Chinese 5-element music therapy (32), logo-autobiography for college students (33), auricular acupressure (34), and mindfulness-based stress reduction (41).

**Measurement tools**. The most common measurement tools used in the studies addressing symptoms included the BDI (31) and the BDI-II (34), the Beck Anxiety Inventory (BAI) (34,36), self-report surveys (37,40), and descriptive statistics (26,29). Other measurement tools included the Coping Styles Inventory (31), the Depression Mood Self-Report Inventory for Adolescence (32), salivary cortisol levels (33), the Pittsburgh Sleep Quality Index (34), the Interpersonal Problem-Solving Inventory (36), and the Premenstrual Syndrome Scale (PMSS) (41).

**Outcomes**. Among the studies included that addressed symptoms, significant improvements were noted post-intervention for depressive symptoms (31–34), stress level (33,37), meaning of life (33), sleep quality (34), anxiety symptoms (34,36,37), PMS symptoms (except swelling) (41), help-seeking for a mental health issue (37,40), and accessing school resources (37). Increases in screening for depression (26,29) amongcollege students were also noted. Chen and colleagues (32) found nonsignificant decreases in salivary cortisol levels, and Tay found no significant differences between the control and intervention groups for barriers to help-seeking behavior (40).

## Discussion

Our literature review underscores substantial differences in the focus of studies in institutions across North America, southeastern Europe, and Asia. Understanding these differences is essential for comprehending the nuanced health care delivery contexts, particularly regarding mental health support for college students. In the US, nurses assume a multifaceted role in collegiate health care settings, acting as primary caregivers, educators, and advocates for students’ well-being (19,21). Their duties include direct care, health promotion, and facilitating access to resources (19,21). Conversely, in global contexts, the role of nurses may fluctuate considerably depending on legislative definitions of nursing practice, educational preparation of nurses, health care systems, cultural norms, and resource availability (46). Nurses may have varying scopes of practice across the globe, where nurses in one country may only be able to practice under the supervision of a physician. In contrast, other countries may recognize the role of advanced practice nursing and a more autonomous role for nurses in patient care, though the scope of practice for advanced practice nurses may also vary (47,^48^). Recognizing these differences is essential for crafting tailored interventions that meet the distinct needs and resources of diverse college communities worldwide.

### Variability of interventions

We found various intervention modalities for addressing the mental health concerns of college students. A stepwise approach was implemented by Aoki and colleagues in a shared decision-making program comprising 3 steps: initial consultation, decision coaching with a nurse, and a decision-making consultation (30). Longer-term interventions included one focused on IRRA, involving 21 sessions lasting 90 minutes each (31). Similarly, Chen and colleagues used the Chinese 5-element music therapy, consisting of 40-minute sessions twice weekly over 10 weeks (32). Another long-term intervention approach was the use of logo-autobiography for college students, where students engaged in autobiographical writing, explored common topics, shared writings with group members, exchanged feedback, and discovered meaning in their lives through 90-minute sessions held weekly for 6 weeks (34).

A unique intervention using auricular acupressure was implemented over 4 weeks and involved the application of a magnetic pellet on the shenmen acupoint of participants (34). The use of technology to implement interventions was another unique feature. These interventions included a mindfulness-based stress reduction program consisting of 8 weekly sessions, including a 6-hour silence retreat conducted via Zoom (41). Similarly, an online mental health promotion program, consisting of 8 individual sessions held twice a week, was supplemented by one-on-one feedback sessions via SMS or messaging applications (38).

Some studies used traditional mental health therapeutic approaches, including cognitive behavioral group (39), group positive psychotherapy (35), mindfulness and yoga (28), and group IRRA (36). Other interventions created education-based programs, including the HOPE intervention, a web-based program consisting of 4 sessions focusing on mental health education (40). Chen and colleagues introduced a nurse-led one-to-one consultation, supplemented by online self-management education modules, over a 12-week period (27). A health education intervention comprising 2 structured visits 2 weeks apart involved structured teachings on various health topics (37). A combination approach was used for a depression screening and referral protocol at a state university health clinic, including education on depression and suicide prevention for all students (26).

The variety of interventions in our review offers valuable insights into the multifaceted strategies employed to overcome mental health issues among college students, highlighting the importance of diverse approaches tailored to individual needs and circumstances.

### Differences in college student populations

Another observation is the diversity in college student populations, both in terms of demographic and clinical characteristics. Studies have indicated differences in the prevalence of mental health disorders among college students across different regions and cultures including Korea (33), Singapore (40), Turkey (31,36,41), and Taiwan (32,34). Additionally, there may be differences between students diagnosed with mental health conditions and those experiencing symptoms without a formal diagnosis. These differences underscore the importance of implementing flexible and inclusive strategies to address the spectrum of mental health needs of college populations. Recognizing these variations enables nurses who care for college students to adopt a holistic approach that encompasses early intervention, symptom management, and support for students at various stages of their academic journey and health care needs. This recognition further highlights the critical role of screening and care coordination in facilitating early detection and intervention for mental health concerns among college students. General health care providers, including nurses, play a pivotal role in implementing systematic screening protocols and identifying students who may be at risk or experiencing mental health symptoms (22,26,29). These clinicians can facilitate timely referrals to mental health professionals, ensuring that students receive appropriate assessment, diagnosis, and treatment, thereby mitigating the potential escalation of mental health challenges. Furthermore, proactive screening and early intervention efforts contribute to destigmatizing mental health issues and fostering a campus culture that prioritizes emotional well-being.

### Depression and anxiety

Rates of depression and anxiety among college students are increasing at an alarming rate (4). An importantfinding of the reviewed studies is the potential for expanding the role of college health nurses in addressing depression and anxiety symptoms among students. Although some interventions focus on students with diagnosed mental health conditions (27,28,30,35,38,39), recognition of the value in extending care to those experiencing symptoms without a formal diagnosis is growing (26,29,31,33,36,37,40,41). Nurse-led interventions, such as counseling, psycho-education, and support groups, have shown promise in providing accessible and stigma-free mental health support (38) with similar findings reported by Amsalem and colleagues and Castillo and colleagues (49,50), and can be replicated in college settings. By broadening the scope of care to encompass symptom management and preventive strategies, college health nurse providers can effectively reach a larger segment of the student population and promote overall psychological resilience. Additionally, state and country regulations may limit the scope of practice for nurses, including advanced practice nurses, which could affect timely access to critical mental health services for college students. Nurses should be able to practice to the full scope of their educational preparation, licensure, and certification.

### Effect of nurse-led interventions on college students' mental health

A recurring theme in the literature is the positive effect of nurse-led interventions on the mental health outcomes of college students. From providing counseling and psychotherapy to promoting wellness and resilience, nurses play a vital role in delivering holistic care that addresses the diverse needs of students. The evidence suggests that nurse-led interventions contribute to improved access to mental health services, reduced stigma (38), and enhanced student engagement in self-care practices (37,38). Moreover, the collaborative nature of nurse-led initiatives can foster a sense of trust and rapport between students and clinicians, facilitating open communication and help-seeking behavior (51).

### Clarification of college versus university terminology

Terminology such as “college” and “university” are often used interchangeably in the US (52), but these terms can have different meanings in educational contexts in other countries. Although in some regions, “college” refers to postsecondary institutions offering undergraduate degrees, “university” usually encompasses broader academic programs, including graduate studies and research. For example, in the United Kingdom, the term “college” refers to either 2-year programs to prepare for university examinations or to vocational programs, whereas “university” or “uni” refers to bachelor’s degree or higher programs (53). Understanding these terminological distinctions is crucial for ensuring clarity and precision in cross-cultural research and intervention efforts to support student mental health globally.

### Limitations

Several studies shared common limitations, including small sample sizes (26,27,29,31,35,38), reliance on self-report data (29,35,41), and the use of convenience sampling methods (28,32,33,38). Furthermore, some studies were limited by their study design, such as being single-center or single-site studies (29,30,33,34,40,41), lacking control groups (33,36), and lacking long-term follow-up (26–29,33,34). Additionally, participant characteristics are sometimes poorly described, such as not specifying the student type (eg, undergraduate, graduate) or the student’s major (39). Moreover, studies often feature predominantly female samples, overlooking the mental health issues in college-aged males. Additionally, the ties to college health centers are noted in a few studies (26,29,30,37), but are not adequately described in others. Furthermore, the small number of countries in which our included studies were conducted does not include most of the world’s geography and may highlight the understudied nature of the nurse’s role in mental health interventions for college students. In addition, because terms such as “college” and “university” have different meanings across the globe, the ability to fully and accurately compare studies is limited. Lastly, our search may not have yielded all articles on nurse-led mental health interventions for college students, particularly if “nurse” or another variation of nurse as noted in our search terms was not included as a key term, indexed as a MeSH term, or in the title or abstract of the article. For example, the Creating Opportunities for Personal Empowerment Program is an evidence-based cognitive–behavioral therapy-based intervention that has been shown to improve symptoms of depression and anxiety among college students in a pilot study but did not appear in our search results because “nurse” was not in the title, abstract, or keywords and was not used in the indexing process (54). Addressing these shared limitations calls for enhanced research methodologies and broader participant inclusion to bolster the generalizability and validity of findings.

### Strengths

Despite these limitations, our findings represent a substantial contribution to the understanding of mental health issues among college students. The reviewed publications offer valuable insights by allowing an examination of mental health challenges in the college/university environment in several areas of the world. This broader outlook enhances the relevance and applicability of the findings to a wide range of college populations worldwide. There is also an opportunity to identify important areas for improvement in campus health practices, particularly in addressing mental health concerns. It is crucial to involve all health care providers, not just mental health specialists, in the school health system in screening and identifying mental health concerns. This comprehensive approach ensures that students receive timely and holistic care, leveraging the expertise of various health care professionals to address their needs effectively. By highlighting common limitations and gaps in the existing literature, our review lays the groundwork for future research and intervention efforts aimed at enhancing mental health support on college campuses.

### Conclusion

Our systematic review highlights the importance and heterogeneity of the nurse’s role in supporting mental health among college students. From mental health screening and care coordination, to delivering targeted and creative mental health interventions, nurses at all levels of practice play a key role in reducing the burden of poor mental health and related symptoms on the college campus. Further research to validate nurse-led mental health intervention effectiveness and policy development are needed to support nursing practice in this understudied role.

## Appendix Search Strategy

**Table:** PubMed

Search number	Query	Results
4	(#1 AND #2 AND #3) AND ((“2015”[Date - Publication]: “3000”[Date - Publication]))	1,000
3	nurses [mh] OR nursing [mh] OR “nurse led” [tiab:~2] OR “nurse managed” [tiab:~2] OR “nurse run” [tiab:~2] OR “nurse directed” [tiab:~2] OR nurse [tiab] OR nurses [tiab] OR “nurse practitioner*” [tiab]	527,492
2	“Student Health Services”[Mesh] OR “Universities”[Mesh] OR “Students”[Mesh] OR “college student” [tiab:~2] OR “university student” [tiab:~2] OR “college students” [tiab:~2] OR “university students” [tiab:~2] OR college [tiab] OR colleges [tiab] OR university [tiab] OR universities [tiab] OR “graduate student” [tiab:~2] OR “graduate students” [tiab:~2] OR “undergraduate student” [tiab:~2] OR “undergraduate students” [tiab:~2] OR “campus health” [tiab:~2] OR “student health” [tiab:~2]	741,684
1	“Anxiety Disorders”[Mesh] OR “Depressive Disorder”[Mesh] OR “Suicide”[Mesh] OR anxiety [tiab] OR anxious [tiab] OR depress* [tiab] OR suicid* [tiab] OR “mood disorder” [tiab:~2] OR “mood disorders” [tiab:~2] OR “disordered mood” [tiab:~2] OR “disordered moods” [tiab:~2]	868,369

**Table:** Embase

No.	Query	Results
#4	#1 AND #2 AND #3 AND [2015–2024]/py	1,245
#3	'nurse'/exp OR 'nursing'/exp OR (((nurse OR nurses OR nursing) NEAR/2 (led OR managed OR run OR directed OR practitioner*)):ti,ab,kw)	616,519
#2	'university'/exp OR 'college'/exp OR 'community college'/exp OR 'school'/de OR 'college student'/exp OR 'graduate student'/exp OR 'phd student'/exp OR 'undergraduate student'/exp OR college*:ti,ab,kw OR universit*:ti,ab,kw OR (((student* OR campus*) NEAR/2 health):ti,ab,kw) OR (((undergrad* OR graduate* OR colleg* OR universt*) NEAR/2 student*):ti,ab,kw)	1,309,306
#1	'anxiety disorder'/exp OR 'anxiety'/exp OR 'depression'/exp OR 'suicidal behavior'/exp OR anxiety:ti,ab,kw OR anxious:ti,ab,kw OR depress*:ti,ab,kw OR suicid*:ti,ab,kw OR ((mood* NEAR/2 disorder*):ti,ab,kw)	1,504,560

Abbreviations: RCT, Randomized Controlled Trial; QI, Quality Improvement.

**Table:** CINAHL

#	Query	Limiters/Expanders	Results
S5	S1 AND S2 AND S3	Limiters - Publication Date: 20150101–20241231 Expanders - Apply related words; Apply equivalent subjects Search modes - Boolean/Phrase	333
S4	S1 AND S2 AND S3	Expanders - Apply related words; Apply equivalent subjects Search modes - Boolean/Phrase	531
S3	(MH “Nurses+”) OR TI ((nurse OR nurses OR nursing) N2 (led OR managed OR run OR directed OR practitioner*)) OR AB ((nurse OR nurses OR nursing) N2 (led OR managed OR run OR directed OR practitioner*))	Expanders - Apply related words; Apply equivalent subjects Search modes - Boolean/Phrase	250,245
S2	(MH “Student Health Services+”) OR (MH “Colleges and Universities+”) OR (MH “Students, College+”) OR (MH “Students, Health Occupations+”) OR TI (college* OR universit* OR (((student* OR campus*) N2 health) OR ((undergrad* OR graduate* OR colleg* OR universit*) N2 student*))) OR AB (college* OR universit* OR (((student* OR campus*) N2 health) OR ((undergrad* OR graduate* OR colleg* OR universit*) N2 student*)))	Expanders - Apply related words; Apply equivalent subjects Search modes - Boolean/Phrase	366,610
S1	(MH “Anxiety Disorders+”) OR (MH “Anxiety+”) OR (MH “Depression+”) OR (MH “Suicide+”) OR TI (anxiety OR anxious OR depress* OR suicid* OR (mood* N2 disorder*)) OR AB (anxiety OR anxious OR depress* OR suicid* OR (mood* N2 disorder*))	Expanders - Apply related words; Apply equivalent subjects Search modes - Boolean/Phrase	358,225

**Table:** APA PsycInfo

#	Query	Limiters/Expanders	Results
S5	S1 AND S2 AND S3	Limiters - Publication Year: 2015–2024 Expanders - Apply related words; Apply equivalent subjects Search modes - Boolean/Phrase	218
S4	S1 AND S2 AND S3	Expanders - Apply related words; Apply equivalent subjects Search modes - Boolean/Phrase	384
S3	DE “Nurses” OR DE “Psychiatric Nurses” OR DE “Public Health Service Nurses” OR DE “School Nurses” OR DE “Nursing” OR TI ((nurse OR nurses OR nursing) N2 (led OR managed OR run OR directed OR practitioner*)) OR AB ((nurse OR nurses OR nursing) N2 (led OR managed OR run OR directed OR practitioner*))	Expanders - Apply related words; Apply equivalent subjects Search modes - Boolean/Phrase	59,400
S2	DE “College Students” OR DE “College Athletes” OR DE “Community College Students” OR DE “Education Students” OR DE “Junior College Students” OR DE “Nursing Students” OR DE “ROTC Students” OR DE “Community College Students” OR DE “Junior College Students” OR DE “Graduate Students” OR DE “Postgraduate Students” OR DE “College Mental Health Services” OR TI (college* OR universit* OR (((student* OR campus*) N2 health) OR ((undergrad* OR graduate* OR colleg* OR universit*) N2 student*))) OR AB (college* OR universit* OR (((student* OR campus*) N2 health) OR ((undergrad* OR graduate* OR colleg* OR universit*) N2 student*)))	Expanders - Apply related words; Apply equivalent subjects Search modes - Boolean/Phrase	383,962
S1	DE “Anxiety” OR DE “Anxiety Disorders” OR DE “Castration Anxiety” OR DE “Generalized Anxiety Disorder” OR DE “Panic Attack” OR DE “Panic Disorder” OR DE “Phobias” OR DE “Selective Mutism” OR DE “Separation Anxiety Disorder” OR DE “Persistent Depressive Disorder” OR DE “Major Depression” OR DE “Anaclitic Depression” OR DE “Dysthymic Disorder” OR DE “Endogenous Depression” OR DE “Late Life Depression” OR DE “Postpartum Depression” OR DE “Reactive Depression” OR DE “Recurrent Depression” OR DE “Treatment Resistant Depression” OR DE “Suicidality” OR DE “Suicidal Behavior” OR DE “Attempted Suicide” OR DE “Suicidal Ideation” OR DE “Suicide” OR DE “Suicide” OR DE “Military Suicide” OR DE “Youth Suicide” OR TI (anxiety OR anxious OR depress* OR suicid* OR (mood* N2 disorder*)) OR AB (anxiety OR anxious OR depress* OR suicid* OR (mood* N2 disorder*))	Expanders - Apply related words; Apply equivalent subjects Search modes - Boolean/Phrase	588,736

**Table:** ERIC

#	Query	Limiters/Expanders	Results
S5	S1 AND S2 AND S3	Limiters - Publication Date: 20150101–20241231 Expanders - Apply related words; Apply equivalent subjects Search modes - Boolean/Phrase	18
S4	S1 AND S2 AND S3	Expanders - Apply related words; Apply equivalent subjects Search modes - Boolean/Phrase	68
S3	DE “Nurses” OR DE “School Nurses” OR DE “Nursing” OR TI ((nurse OR nurses OR nursing) N2 (led OR managed OR run OR directed OR practitioner*)) OR AB ((nurse OR nurses OR nursing) N2 (led OR managed OR run OR directed OR practitioner*))	Expanders - Apply related words; Apply equivalent subjects Search modes - Boolean/Phrase	6,717
S2	DE “School Health Services” OR DE “Noncampus Colleges” OR DE “Private Colleges” OR DE “For Profit Colleges” OR DE “Public Colleges” OR DE “Community Colleges” OR DE “State Colleges” OR DE “Religious Colleges” OR DE “Single Sex Colleges” OR DE “Small Colleges” OR DE “Two Year Colleges” OR DE “Multicampus Colleges” OR DE “Upper Division Colleges” OR DE “Commuting Students” OR DE “College Students” OR DE “College Freshmen” OR DE “College Seniors” OR DE “College Transfer Students” OR DE “First Generation College Students” OR DE “Graduate Students” OR DE “In State Students” OR DE “On Campus Students” OR DE “Out of State Students” OR DE “Preservice Teachers” OR DE “Two Year College Students” OR DE “Undergraduate Students” OR DE “Graduate Students” OR DE “Doctoral Students” OR DE “Law Students” OR DE “Medical Students” OR DE “Two Year College Students” OR DE “Community College Students” OR DE “Undergraduate Students” OR DE “Premedical Students” OR DE “Colleges” OR DE “Universities” OR DE “Land Grant Universities” OR DE “Open Universities” OR DE “Research Universities” OR DE “State Universities” OR DE “Urban Universities” OR TI (college* OR universit* OR (((student* OR campus*) N2 health) OR ((undergrad* OR graduate* OR colleg* OR universit*) N2 student*))) OR AB (college* OR universit* OR (((student* OR campus*) N2 health) OR ((undergrad* OR graduate* OR colleg* OR universit*) N2 student*)))	Expanders - Apply related words; Apply equivalent subjects Search modes - Boolean/Phrase	498,163
S1	DE “Anxiety” OR DE “Anxiety Disorders” OR DE “Posttraumatic Stress Disorder” OR DE “Depression (Psychology)” OR DE “Suicide” OR TI (anxiety OR anxious OR depress* OR suicid* OR (mood* N2 disorder*)) OR AB (anxiety OR anxious OR depress* OR suicid* OR (mood* N2 disorder*))	Expanders - Apply related words; Apply equivalent subjects Search modes - Boolean/Phrase	44,670
